# Endogenous Viral Elements in Shrew Genomes Provide Insights into *Pestivirus* Ancient History

**DOI:** 10.1093/molbev/msac190

**Published:** 2022-09-05

**Authors:** Yiqiao Li, Magda Bletsa, Zafeiro Zisi, Ine Boonen, Sophie Gryseels, Liana Kafetzopoulou, Joanne P Webster, Stefano Catalano, Oliver G Pybus, Frederik Van de Perre, Haotian Li, Yaoyao Li, Yuchun Li, Alexei Abramov, Petros Lymberakis, Philippe Lemey, Sébastian Lequime

**Affiliations:** Department of Microbiology, Immunology and Transplantation, Rega Institute, KU Leuven, 3000 Leuven, Belgium; Department of Microbiology, Immunology and Transplantation, Rega Institute, KU Leuven, 3000 Leuven, Belgium; Department of Microbiology, Immunology and Transplantation, Rega Institute, KU Leuven, 3000 Leuven, Belgium; Department of Microbiology, Immunology and Transplantation, Rega Institute, KU Leuven, 3000 Leuven, Belgium; Department of Microbiology, Immunology and Transplantation, Rega Institute, KU Leuven, 3000 Leuven, Belgium; Belgium Evolutionary Ecology Group, University of Antwerp, 2610 Wilrijk, Belgium; Department of Microbiology, Immunology and Transplantation, Rega Institute, KU Leuven, 3000 Leuven, Belgium; Virology Department, Belgium Bernhard Nocht Institute for Tropical Medicine, 20359 Hamburg, Germany; Department of Pathobiology and Population Science, Royal Veterinary College, University of London, Herts, AL9 7TA, UK; Department of Pathobiology and Population Science, Royal Veterinary College, University of London, Herts, AL9 7TA, UK; Department of Pathobiology and Population Science, Royal Veterinary College, University of London, Herts, AL9 7TA, UK; Belgium Evolutionary Ecology Group, University of Antwerp, 2610 Wilrijk, Belgium; Marine College, Shandong University (Weihai), 264209 Weihai, China; Marine College, Shandong University (Weihai), 264209 Weihai, China; Marine College, Shandong University (Weihai), 264209 Weihai, China; Laboratory of Theriology, Zoological Institute of the Russian Academy of Sciences, 190121 Saint Petersburg, Russia; Natural History Museum of Crete, Iraklio 712 02, Greece; Department of Microbiology, Immunology and Transplantation, Rega Institute, KU Leuven, 3000 Leuven, Belgium; Department of Microbiology, Immunology and Transplantation, Rega Institute, KU Leuven, 3000 Leuven, Belgium; Cluster of Microbial Ecology, Groningen Institute for Evolutionary Life Sciences, University of Groningen, 9747 AG Groningen, the Netherlands

**Keywords:** endogenous viral element, pestivirus, *Flaviviridae*, *Crocidura*, host range, paleovirology

## Abstract

As viral genomic imprints in host genomes, endogenous viral elements (EVEs) shed light on the deep evolutionary history of viruses, ancestral host ranges, and ancient viral–host interactions. In addition, they may provide crucial information for calibrating viral evolutionary timescales. In this study, we conducted a comprehensive *in silico* screening of a large data set of available mammalian genomes for EVEs deriving from members of the viral family *Flaviviridae*, an important group of viruses including well-known human pathogens, such as Zika, dengue, or hepatitis C viruses. We identified two novel pestivirus-like EVEs in the reference genome of the Indochinese shrew (*Crocidura indochinensis*). Homologs of these novel EVEs were subsequently detected *in vivo* by molecular detection and sequencing in 27 shrew species, including 26 species representing a wide distribution within the Crocidurinae subfamily and one in the Soricinae subfamily on different continents. Based on this wide distribution, we estimate that the integration event occurred before the last common ancestor of the subfamily, about 10.8 million years ago, attesting to an ancient origin of pestiviruses and *Flaviviridae* in general. Moreover, we provide the first description of *Flaviviridae*-derived EVEs in mammals even though the family encompasses numerous mammal-infecting members. This also suggests that shrews were past and perhaps also current natural reservoirs of pestiviruses. Taken together, our results expand the current known *Pestivirus* host range and provide novel insight into the ancient evolutionary history of pestiviruses and the *Flaviviridae* family in general.

## Introduction

Endogenous viral elements (EVEs) are integrations of partial or full-length viral genomic material into the host genome (Katzourakis and Gifford 2010). In addition to retroviruses, which incorporate their genomic sequences into their host genome as an essential part of their replication cycle, many eukaryotic viruses have been found endogenized in various hosts (Feschotte and Gilbert 2012). These non-retroviruses can derive from dsDNA (Aswad and Katzourakis 2014; Li and Li 2015; Liu *et al*. 2020), ssDNA (Belyi *et al*. 2010a; Katzourakis and Gifford 2010; Kobayashi *et al*. 2019), dsRNA (Horie *et al*. 2010; Katzourakis and Gifford 2010; Liu *et al*. 2012), and even ssRNA viruses (Crochu *et al*. 2004; Horie *et al*. 2010; Lequime and Lambrechts 2017; Flynn and Moreau 2019). Non-retroviral RNA virus-derived EVEs arise from a conjunction of relatively rare events: (i) production of DNA genomic material, using retrotransposon encoded reverse transcriptase (Horie *et al*. 2010; Horie 2019); (ii) integration in the host chromosome of germ line cells; and (iii) overcoming genetic drift and/or natural selection at the population until fixation (Holmes 2011; Aiewsakun and Katzourakis 2015). EVEs thus reflect long-term and intimate interactions of viruses with their hosts, and their identification can reveal insights into past and present host distributions of viral genera and families. The detection of endogenous bornavirus-like elements in invertebrate genomes (Horie *et al*. 2013), for example, suggested a broader host range for bornaviruses than previously thought. The study of filovirus-like EVEs in some small mammals offer predictive value for further identifying filovirus reservoirs (Taylor *et al*. 2010). Similarly, the discovery of flavivirus-derived EVEs in *Anopheles* mosquito genomes supported the idea that *Anopheles* mosquitoes could also be natural hosts of flaviviruses (Lequime and Lambrechts 2017), as confirmed by other studies (Colmant *et al*. 2017; Öncü *et al*. 2018).

Aside from qualitative insights into host ranges of viruses, EVEs can also shed light on deep evolutionary histories of viruses. EVEs are significant traces of past virus–host interactions; unlike animals or plants, viruses do not leave physical fossil records, limiting our ability to study their deep evolutionary histories. EVEs could thus be considered as ‘genomic fossil records’ that can help to unravel long-term evolutionary dynamics between hosts and viral families. The presence of EVE homologs in different host species hints at an integration event before speciation, here the time to the most recent common ancestor. It thus provides a minimum age estimate for the integration event, and therefore a minimum age for the existence of a specific viral taxonomic group (Aiewsakun and Katzourakis 2015). For example, EVEs derived from adeno-associated viruses appear to be orthologous in African and Asian elephants, indicating an integration event more than 6 million years ago (Kobayashi *et al*. 2019). Similarly, the discovery of abundant bornavirus-like EVEs across vertebrates reveals that ancient bornaviral infections occurred over a timeframe of about 100 million years before present (Kawasaki *et al*. 2021). These studies can also provide genetic fossil calibration points for further gauging ancient viral timescales using phylogenetics (Feschotte and Gilbert 2012).

Members of the *Flaviviridae* family are linear, positive-sense, single-stranded RNA viruses currently classified in four recognized genera (*Flavivirus*, *Hepacivirus*, *Pestivirus*, and *Pegivirus*). They encompass many significant pathogens, such as dengue, Zika, hepatitis C, or bovine viral diarrhea virus. In addition to human and livestock infections, *Flaviviridae* viruses have been detected in a broad range of mammalian wildlife hosts, e.g., nonhuman primates (Mares-Guia *et al*. 2020), rodents (Bletsa *et al*. 2021), bats (Wu *et al*. 2018), but also birds (Strand *et al*. 2018), fish (Hartlage 2016; Soto *et al*. 2020), and arthropods (Shi *et al*. 2016). Despite this important host range, current published studies have only identified *Flaviviridae*-derived EVEs in arthropods, including mosquitos (Crochu *et al*. 2004; Roiz *et al*. 2009; Lequime and Lambrechts 2017), ticks (Maruyama *et al*. 2014), and crustaceans (Parry and Asgari 2019), and these are all related to the *Flavivirus* genus. Currently, however, convincing evidence for *Flaviviridae*-derived EVEs in vertebrates remains lacking. Interestingly, potential integration has been suggested in medaka fish (Belyi et al. 2010b) as well as in rabbit and hare genomes (Silva et al. 2012, 2015). While this raises the hypothesis that *Flaviviridae* viruses have integrated in vertebrate hosts, the origin of these specific genomic sequences remains inconclusive due to their short size and low sequence similarity to the *Flaviviridae* (*Flavivirus* and *Hepacivirus,* respectively). A recent unpublished study also identified *Flaviviridae*-derived EVEs in a wide variety of hosts, including various invertebrates and fish (Bamford *et al*. 2021).

In this study, we explored *in silico* the presence of *Flaviviridae*-derived EVEs in a comprehensive set of mammalian genomes, and we discovered two novel pesti-like EVEs in the genome of the Indochinese shrew *Crocidura indochinensis.* We subsequently identified and characterized homologs of these EVEs *in vivo* in 26 species of the Crocidurinae subfamily and one member of the Soricinae subfamily, establishing the integration event at least 10.8 million years ago. Our results provide the first evidence for an ancient origin of pestiviruses and also contribute to a better understanding of the evolutionary history of the *Flaviviridae* family in general.

## Results

### False Positive Flaviviridae-Like Hits from Mammalian Genome Screening

Our initial screening of 689 available mammalian genomes using *Flaviviridae* and *Flaviviridae*-like polyproteins yielded 66 positive hits from 49 species, including rodents, nonhuman primates, marsupials, insectivores, carnivores, and bats. Detailed information about our *in silico* screening results can be found in Supplementary Table S1, Supplementary Material online. All virus-related sequences aligned with three pestiviruses, namely border disease virus, bovine viral diarrhea virus 1, and bovine viral diarrhea virus 2. With the exception of one shrew species (see section below), the position of all hits in the corresponding viral genomic sequence was in the ubiquitin-homolog domain between the nonstructural proteins NS2 and NS3, while some of the hits slightly expanded the alignment to the NS3 region (shown in Supplementary Fig. S1, Supplementary Material online). The ubiquitin domain in some bovine viral diarrhea virus strains is however predicted to originate from cellular derived insertions in cytopathogenic pestivirus (Agapov *et al*. 2004; Becher and Tautz 2011). The similarity between this viral genomic region and ubiquitin poses a considerable risk for false positives when searching for pestivirus-derived EVEs, and these hits were therefore not further considered.

### *In Silico* Identification of *Crocidura indochinensis* Pesti-Like EVEs

**Fig. 1.**
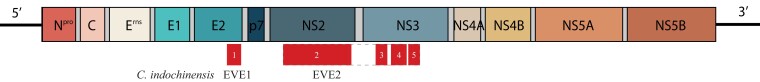
The positions of the newly detected *Crocidura indochinensis* EVEs are shown relative to an archetypal *Pestivirus* genome (classical swine fever virus, NC_002657). N^pro^, N-terminal protease; C, nucleocapsid core protein; E^rns^, envelope glycoprotein E^rns^; E1, envelope glycoprotein E1; E2, envelope glycoprotein E2; p7, nonstructural protein p7; NS2, nonstructural protein NS2; NS3, nonstructural protein NS3; NS4*A*, nonstructural protein NS4*A*; NS4*B*, nonstructural protein NS4*B*; NS5*A*, nonstructural protein NS5*A*; NS5*B*, nonstructural protein NS5*B*.

Besides the ubiquitin-related false positive results, our *in silico* screening identified a series of five *Flaviviridae*-related EVEs fragments in a single contig of the *Crocidura indochinensis* reference genome PVKC01 (Table 1). The first EVE (EVE1) is 318 nt long, with its closest BLAST hit being the Linda virus (*Pestivirus*) envelope glycoprotein E2 region (tBLASTx, 25.5% identity, e-value 3.31E^−35^), without any stop codon (Fig. 1). The remaining four EVEs fragments are 1,053, 84, 114 and 87 nt long, respectively, with their closest BLAST hits being a classical swine fever virus and a rodent pestivirus (with minimum amino acid identity 24.9%, maximum 62.1%). These four fragments are separated by very short gaps, with lengths of 16, 1, and 4 nucleotides, respectively. The two central fragments of EVE2, fragments 3 (84 nt), and fragment 4 (114 nt), are in a different translation frame than the two others but in the same orientation. Their arrangement in the host contig reflects their relative position in the pestiviral genome, which partially spans the nonstructural NS2 and NS3 genes (Fig. 1). For these reasons, in further analyses, we considered these four fragments as the result of a single pestiviral integration event, and thus a unique EVE (EVE2). Phylogenetic reconstructions of the identified *Crocidura indochinensis* EVE1, EVE2, and entire concatenated sequence with exogenous *Flaviviridae* viruses supports the pestivirus-origin of the EVEs (Supplementary Fig. S2, Supplementary Material online).

**Table 1.** Newly Detected EVEs in *Crocidura Indochinensis* Genom**e.**

Contig accession no.	Contig length (nt)	EVE	GenBank accession no.	EVEs fragments	EVE length (nt)	Position in host contig		Translation frame	Closest BLAST hit	Conserved domain search
						Start	End			
PVKC010097735.1	6104	EVE1	BK014483	No. 1	318	2329	2646	1	Linda virus	Pestivirus envelope glycoprotein E2
		EVE2		No. 2	1053	3205	4257	1	Classical swine fever virus	Peptidase_C74: Pestivirus NS2 peptidase
				No. 3	84	4274	4357	2	Rodent pestivirus	Peptidase_S31: Pestivirus NS3 polyprotein peptidase S31
				No. 4	114	4359	4472	2	Rodent pestivirus	Peptidase_S31: Pestivirus NS3 polyprotein peptidase S31
				No. 5	87	4477	4563	1	Rodent pestivirus	Peptidase_S31: Pestivirus NS3 polyprotein peptidase S31

In addition, a fragment prior to EVE1 in the contig shows a strong similarity (tBLASTx, 45.2% identity, e-value 5.51E^−19^, supplementary Table S1, Supplementary Material online) with the pestivirus ribonuclease T2 gene. However, considering that this enzyme exists in a wide range of organisms (Luhtala and Parker 2010), the virus-derived origin of this sequence in *Crocidura indochinensis* is not guaranteed.

The position of all pestivirus-like hits, including the ribonuclease T2 gene, in the host contig corresponds to their relative organization in the pestivirus genome. No additional features were detected after a tBLASTx search of the whole contig encompassing the two identified EVEs (Supplementary Table S1, Supplementary Material online). In addition, we did not detect the EVE sequences in reads of publicly available experimental genomic and transcriptomic data from Soricidae species.

### Identification and Distribution of Pesti-Like EVEs in Other Soricidae Species

To expand our screening and evaluate the distribution of these newly identified EVEs in additional species that are phylogenetically close to *Crocidura indochinensis*, we undertook a PCR-based screening of 65 samples from 29 species of the Soricidae family (Supplementary Table S2, Supplementary Material online). These samples belonged to seven different genera (*Crocidura*, *Paracrocidura*, *Scutisorex*, *Suncus*, *Sylvisorex*, *Neomys,* and *Sorex*), encompassing two subfamilies, Crocidurinae and Soricinae. Cytochrome b (CYTB) genomic sequences were also generated to confirm the species identification (Supplementary Table S3 and Supplementary Fig. S3, Supplementary Material online).

**Fig. 2.**
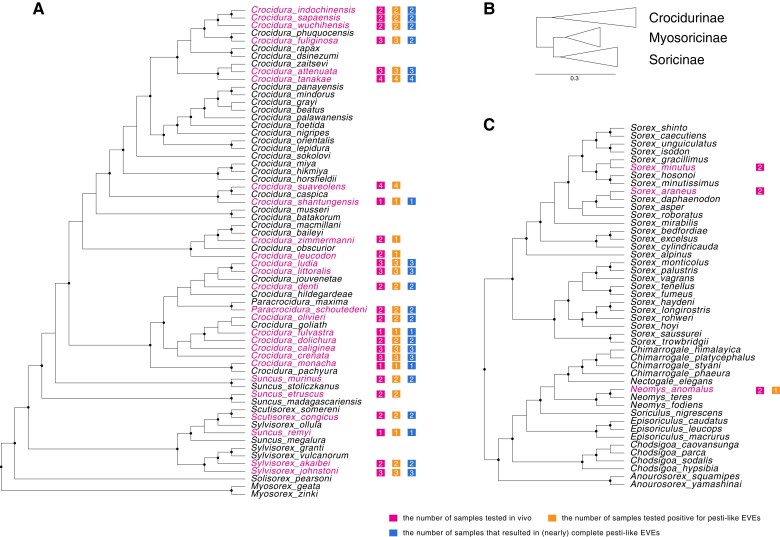
Maximum likelihood phylogeny of the Soricidae (shrews) family based on the dataset of 31 assembled nuclear & mitochondrial genes from (Upham *et al*. 2019). For the four species (*Crocidura denti*, *Crocidura sapaensis*, *Scutisorex congicus*, *Sylvisorex akaibei*) tested in this study and not included in the Upham *et al*. 2019 data set, we used our generated CYTB sequences in the alignment. Nodes labeled in black circles indicate Shimodaira-Hasegawa (SH)-like branch support (%, only values > 80% are shown). The available samples’ species distribution in this study were highlighted in pink: (*A*) The phylogeny of Crocidurinae subfamily and sample distribution; (*B*) Subfamilies relationships within the Soricidae family; (*C*) The phylogeny of Soricinae subfamily and sample distribution.

**Fig. 3.**
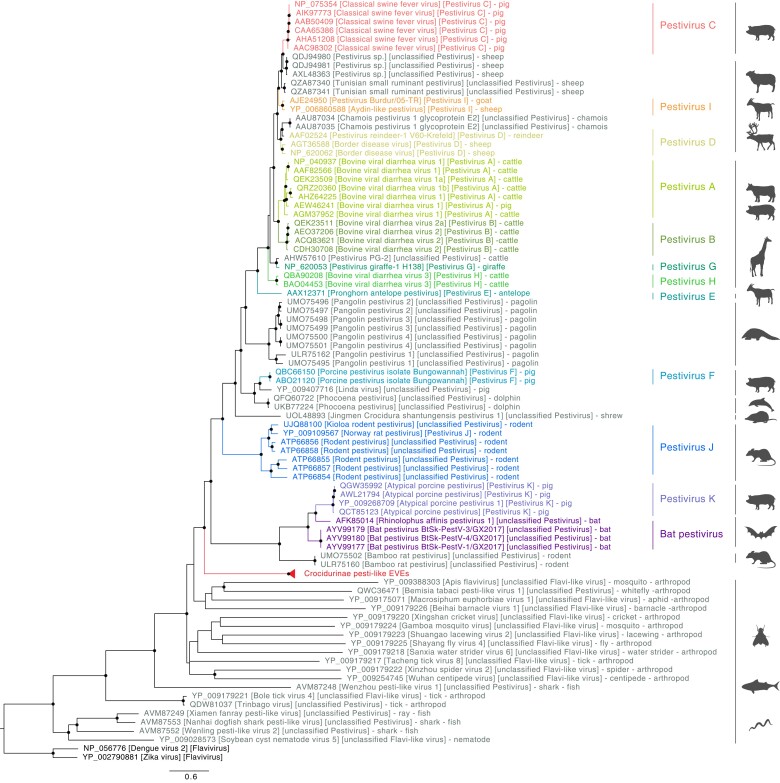
Phylogenetic relationships of pesti-like EVEs with representative *Pestivirus* species and pesti-like viruses (based on aligned viral E2 and NS2-3 region). Dengue and Zika virus (*Flavivirus*) are used as outgroup. Clades are colored based on viral species. Nodes labeled in black circles indicate Shimodaira-Hasegawa (SH)-like branch support (%, only values > 80% are shown). Scale bars indicate the number of amino acid substitutions per site.

In total, 58 samples contained the newly identified pesti-like EVEs, representing 26 species in the Crocidurinae subfamily and one species (*Neomys anomalus*) in the Soricinae subfamily. Among them, 48 samples from 22 species in the Crocidurinae subfamily yielded complete or nearly complete EVEs sequences (Fig. 2). All novel EVEs sequences were highly similar to the *Crocidura indochinensis* EVEs sequences, with a mean identity of 90.38% on amino acid level and 95.12% on nucleotide level, respectively (Supplementary Table S4, Supplementary Material online). Phylogenetic reconstruction indicated that all Crocidurinae pesti-like EVEs clustered together as a sister-lineage of currently recognized *Pestivirus* species but divergent from the currently sole available Crocidurinae pestivirus (Fig. 3, Supplementary Fig. S4, Supplementary Material online).

Although collected in different locations, nearly all species tested in the Crocidurinae subfamily harbored the pesti-like EVEs sequences. For some species, however, such as *Crocidura* cf. *zimmermanni*, *C. leucodon*, *C. suaveolens,* and *Suncus etruscus*, we could not always detect or sequence the EVEs in all samples. The failed detection could be explained by the genomic template being of poor quality due to storage conditions associated with the museum specimens. Interestingly, the pesti-like EVEs were also detected in one *Neomys anomalus* sample, while the remaining Soricinae specimens yielded negative results. The widespread nature of these homolog EVEs in the Crocidurinae species suggests a single endogenization event before their common ancestor about 10.8 million years ago (Dubey *et al*. 2007). Since we were not able to sequence the complete pesti-like EVEs from the *Neomys anomalus* specimen, their phylogenetic relationship to the Crocidurinae pesti-like EVEs remain unclear and they may not necessarily derive from the same endogenization event.

**Fig. 4.**
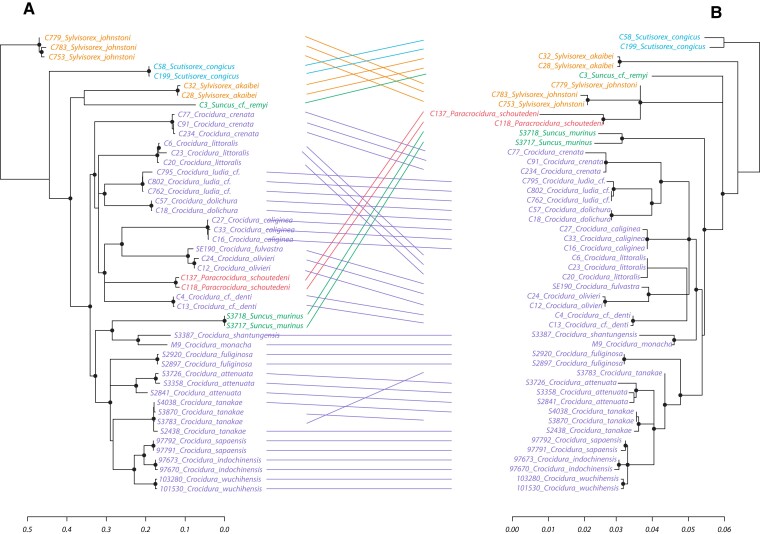
Tanglegram of the cytochrome b phylogeny (*A*) and the corresponding EVEs phylogeny (*B*). The cytochrome b tree was inferred for gene sequences from 22 shrew species, and the EVEs tree (right) was inferred using the newly generated EVEs sequences. The clades were colored by shrew genus, orange: *Sylvisorex*; blue: *Scutisorex*; green: *Suncus;* purple: *Crocidura*; red: *Paracrocidura*. The clade nodes with SH-like branch support < 50% were collapsed as polytomies. The black circles indicate the SH-like branch support > 80%. Lines connect corresponding tips in the two phylogenies. Scale bars indicate the number of nucleotide substitutions per site.

To assess phylogenetic congruence between the EVEs and the shrew host genomes, we performed reconciliation analyses for the evolutionary history of the EVEs with two nuclear and one mitochondrial gene for the available shrew species. All comparisons (EVEs-APOB, EVEs-BRCA1, EVEs-CYTB, CYTB-APOB, CYTB-BRCA1, and APOB-BRCA1) resulted in statistically significant co-phylogenetic patterns with *P*-values < 0.01. Despite the limited degree of phylogenetic variability observed in some co-phylogenetic plots, overall, the EVEs exhibit a pattern of inheritance as any other host genetic markers and follow the evolutionary history of the shrew host species (Fig. 4, Supplementary Fig. S5, Supplementary Material online).

### Selective Pressure on Pesti-Like EVEs

To evaluate potential functional constraints on the EVE, we performed selective pressure analyses. Specifically, we measured selective pressure by estimating the ratio of nonsynonymous substitution rate (*d_N_*) and synonymous substitutions rate (*d_S_*) in protein-coding sequences using two methods: (i) fixed effects likelihood (FEL) (Kosakovsky Pond and Frost 2005); and (ii) Bayesian renaissance counting (Lemey *et al*. 2012). It is expected that nonfunctional regions should conform to neutral evolution, whereas functional regions should experience purifying selection. Based on 48 potentially coding sequences for pesti-like EVE1, the FEL method indicates an overall neutral evolution with *d_N_*/*d_S_* = 0.94. Likewise, the Bayesian renaissance counting model yields a ratio at 1.10 (95% credible interval: 0.66, 1.62), reflecting neutral evolution. The EVE2 coding region was also evaluated using the same methods resulting in a *d_N_*/*d_S_* ratio of 0.77 using FEL and 0.74 (95% credible interval: 0.58, 0.90) using renaissance counting. While the EVE2 estimates appear to reject strict neutrality for EVE2, the *d_N_*/*d_S_* estimates remain relatively high and hence do not suggest strong purifying selection. Moreover, the multiple stop codons and frame-shifts in EVE2 sequences strongly support the absence of functional constraints.

### Towards a Pestivirus Evolutionary Timescale

Having established that the pesti-like EVE arose through a single endogenization event before the Crocidurinae common ancestor offers an opportunity to gain insights into the timescale of pestiviruses. However, the evolutionary history of pestiviruses and pesti-like EVEs (Fig. 3) represents a complex scenario that combines viral evolution over long time scales, which is known to be subject to time-dependent evolutionary rates (Duchêne *et al*. 2014; Aiewsakun and Katzourakis 2016, Membrebe *et al*. 2019), and EVE evolution at the host evolutionary rate. In the absence of molecular clock models that can accommodate such scenarios, we performed a naive divergence time estimation using a standard relaxed molecular clock model and using the estimated Crocidurinae the time to most recent common ancestor (TMRCA) as a calibration on the EVE TMRCA (cfr. Methods). This results in a TMRCA of pestivirus species of 231 (95% HPD: 148–329) MYA, a TMRCA of all pestiviruses and pesti-like viruses of 453 (95% HPD: 296–674) MYA, and a TMRCA of the latter and two flavirus outgroups of 472 (95% HPD: 297–702) MYA.

## Discussion

Non-retroviral EVEs are rare traces of the ancient evolutionary history of viruses. These genomic fossils offer valuable insights into host range, ancestral genetic diversity and can provide invaluable information for dating viral evolutionary history (Feschotte and Gilbert 2012; Aiewsakun and Katzourakis 2015). In our study, we screened a comprehensive set of mammalian genomes to discover such *Flaviviridae*-derived EVEs. We uncovered two *Flaviviridae*-derived EVEs sequences in the genome of the Indochinese shrew and confirmed their presence in a broad range of shrew species belonging to the Crocidurinae subfamily.

The EVEs we identified are related to extant viruses within the *Pestivirus* genus. Viruses belonging to this genus were initially detected in a variety of artiodactylous hosts, such as ruminants and swine, in which they cause subclinical or clinical infections including hemorrhagic syndrome, abortion, acute fatal mucosal disease. Recent metagenomic studies extended the host range towards rodents (Wu et al. 2018, 2020), bats (Wu *et al*. 2018), fish (Shi *et al*. 2018), and ticks (Sameroff *et al*. 2019), but to some extent, the restricted sampling beyond agriculturally important animals limits our understanding of the real host range. Shrews, for example, have been recently identified as hosts of hepaciviruses, another genus in the *Flaviviridae* family (Guo *et al*. 2019; Wu *et al*. 2020), but limited evidence shows its relationship with pestivirus. The broad detection of pestivirus-derived EVEs reported in our study strongly supports that ancestors of the Crocidurinae shrew subfamily have been hosts of pestiviruses and suggests that their descendants might still be. Indeed, considering the extremely low probability of a non-retroviral endogenization event to occur in the germline, EVEs are strong indicators of frequent interactions between the original exogenous viruses and their hosts (Feschotte and Gilbert 2012; Aiewsakun and Katzourakis 2015). This hypothesis is supported by a recent directly submitted sequence in National Center for Biotechnology Information (NCBI) originating from a Chinese *Crocidura shantungensis* shrew, strongly suggesting the presence of exogenous pestivirus in Crocidurinae shrew. Interestingly, the newly described EVE sequences are not directly related to this Crocidura pestivirus. The difference might be due to the genetic drift since the historical endogenization event, or the endogenization of another, currently undetected shrew pestivirus clade. Our study thus provides indirect support for a wider and more diverse host range of pestiviruses. Additional efforts for direct detection and characterization of pestiviruses from shrews would still be required to formally demonstrate the natural hosts range of pestiviruses and to characterize the relationship between exogenous pestivirus and pesti-like EVEs.

Given the low probability of endogenization events of non-retroviral RNA viruses and the contiguous nature of the two EVEs on the host and viral genome, our results suggest a single endogenization event followed by genetic drift. One or several insertion events separated the original EVE in two fragments, EVE1 and EVE2. EVE1 shows a short but intact open reading frame to be evolving under neutral evolution while EVE2 exhibits multiple stop codons and frame-shifts due to additional insertions. Many studies have identified the important roles that EVEs can play in host antiviral immunity, both in vertebrates and invertebrates (Ophinni *et al*. 2019; Blair *et al*. 2020; Skirmuntt *et al*. 2020). Flavivirus-like EVEs in *Aedes* mosquitoes, for example, can produce P-element-induced wimpy testis (PIWI)-interacting RNAs (piRNAs) which limit the cognate virus replication (Suzuki *et al*. 2020). It is highly unlikely that the pesti-like sequences we discovered currently have a function in shrews because of the absence of negative selection and the disruption of the original viral coding region. It does not, however, exclude that the pesti-like sequences may have served a function in the past, following their integration, but we currently have no evidence to support this hypothesis.

The evolutionary history of the EVEs sequences after integration largely follows the host genetic patterns of inheritance. Despite showing significant phylogenetic consistency with the host mitochondrial and nuclear gene sequences (*P*-value < 0.01), there is a limited degree of discordance, particularly in the co-phylogenetic plot of CYTB and the EVEs phylogeny (Fig. 4). These discrepancies might be explained by differences in the genetic inheritance of the CYTB gene and EVEs: the CYTB gene is a mitochondrial gene whereas EVEs are integrated in the nuclear genome. Moreover, discordances are highlighted, although to a lesser extent, in the reconciliations of the APOB and BRCA1 nuclear genes with the EVEs phylogeny, as well as in the co-phylogenetic plot of the CYTB with the nuclear genes (Supplementary Fig. S5, Supplementary Material online). These results are in line with the different observed evolutionary patterns between host genetic markers, especially in the case of weak reproductive isolation within species or species complex, allowing hybridization, as has been suggested for some *Crocidura* species (Vogel et al. 2004; Dubey et al. 2006, 2008).

Dating the ancient evolutionary history of ssRNA viruses such as pestiviruses and *Flaviviridae* in general is challenging. The most commonly used method for inferring viral divergence time is based on the estimation of evolutionary rates derived from sequence data and their collection dates. However, the applicability of this method is often limited by different rate estimates on different timescales (Aiewsakun and Katzourakis 2016) and rate variation among viral lineages (Duffy *et al*. 2008; Sanjuán 2012). Not accounting for the former leads to recent estimates for the origins of ssRNA viruses that are often in conflict with other phylogenetic evidence (Holmes 2003). Using suitable molecular clock models, the powerful combination of both tip and node calibrations may help to recover more accurate evolutionary timescales (O’Reilly *et al*. 2015). Node calibration is however challenging for viruses as no fossil evidence can be found. It thus often relies on known phylogeographic events and other indirect calibrations point, such as ecological events or assumptions of co-divergence as alternative (Bamford *et al*. 2021; Moureau *et al*. 2015; Pettersson and Fiz-Palacios 2014). The discovery of ssRNA virus-related EVEs, thus, offers opportunities for estimating deep timescales of virus evolution history by co-phylogenetic analysis of EVE’s orthologs in different hosts (Gilbert and Feschotte 2010). However, when combining EVEs and extant viruses, we need to consider both EVE evolution according to the host evolutionary rate and time-dependent viral evolutionary rates.

The pesti-like EVE sequences characterized in our study are widespread in Crocidurinae species, are monophyletic and exhibit high sequence similarity. Considering the low probability of endogenization events of non-retroviral RNA viruses, this suggests that the pesti-like EVE got integrated before the most recent common ancestor of the subfamily, which is estimated to be over 10.8 million years ago (Dubey *et al*. 2007). There are only a handful of molecular dating estimates for pestiviruses, and they mostly focus on viral species or clades that are associated with economic losses. Diversification of bovine viral diarrhea virus 1 (Pestivirus A) subtypes was estimated to have started about 363 years ago (Weber *et al*. 2021), and the divergence of HoBi-like pestivirus (Pestivirus H) was dated back to the 16th century (Silveira *et al*. 2020). Applying the Crocidurinae TMRCA as a calibration, we estimate that the pestiviruses date back several hundreds of MY ago. However, in the absence of molecular clock models that can tackle complex virus and EVE evolutionary histories, we present our estimates using a standard relaxed molecular clock as naive estimates. Considering that a recent mechanistic model of time-dependent rates assumes that the long-term viral rate aligns to the host evolutionary rate (Ghafari *et al*. 2021), and that our EVE calibration relies on the host evolutionary rate, it may be that the deep divergence estimates still provide a reasonable indication of the age of pestiviruses (and flaviviruses). Another study estimates, based on a strong co-divergence hypothesis, emergence of pestiviruses at 465 MY ago (Bamford *et al*. 2021). While exact dating between our naive estimates and another study somewhat diverges, both strongly support an ancient evolutionary history of these viruses going back at least several hundred MY ago.

In conclusion, we discovered and characterized the first *Flaviviridae*-related EVEs records from mammalian reference genomes, which derived from pestiviruses. The wide EVEs distribution in shrew Crocidurinae subfamily indicates they are a historical host group of pestiviruses and further suggests an ancient origin time of the *Pestivirus* genus. Our results show the key role of EVEs not only in expanding our knowledge about ancient viral–host interactions, but also their importance in reconstructing the viral evolutionary history, which contributes to our understanding of viral evolutionary dynamics from ancient times to the present.

## Materials and Methods

### In Silico Survey

#### Data Collection

To screen for *Flaviviridae*-like EVEs, 689 mammalian genomes (57 bats, 9 insectivores, 177 rodents, 101 nonhuman primates, 207 even-toed ungulates, 15 odd-toed ungulates, 108 carnivores, and 15 marsupials), were retrieved from the NCBI Whole Genome Shotgun (WGS) database (last accessed in November 2020). A detailed list of all the surveyed mammalian genomes is provided in Supplementary Table S5, Supplementary Material online. A representative group of 306 *Flaviviridae* or *Flaviviridae*-like polyprotein sequences was compiled from the NCBI non-redundant protein database (accessed in February 2019). We provide a list of the nucleotide/protein accession numbers in Supplementary Table S6, Supplementary Material online.

#### Genome Screening

*Flaviviridae* polyprotein sequences were used as queries in tBLASTn (BLAST+ v2.6.0) (Camacho *et al*. 2009) searches with mammalian genomes as targets. We only considered hits with an E-value < 10^−4^. BLAST hits from the same contig and same orientation with gap size < 100 nt were combined into a single fragment, due to the great possibility of sharing the same integration event and to increase the computational efficiency. To avoid potential artifacts, only the hits with length ≥ 250 nt were extracted from mammalian genomes based on the reported position by BLAST in the host contig. These putative EVEs were then used as query in a reciprocal tBLASTx (BLAST+ v2.6.0) (Camacho *et al*. 2009) against a local NCBI nucleotide (nt) database (accessed in October 2018) and BLASTx (BLAST+ v2.6.0) (Camacho *et al*. 2009) against a non-redundant protein (nr) database (accessed in October 2018). EVEs were confirmed if the best hits contained *Flaviviridae* family members with an E-value < 10^−4^. The presence of conserved viral genetic features within the hits was assessed using the NCBI Conserved Domain Database (Marchler-Bauer *et al*. 2015).

#### EVE Characterization

Upon identification of the EVEs, they were translated and aligned with corresponding polyprotein sequences from several representative *Flaviviridae* species using MAFFT v7.453 (Katoh *et al*. 2002). All alignments were trimmed in BMGE v1.12 (Criscuolo and Gribaldo 2010) in order to select for phylogenetic informative regions. The best substitution models were PMB + G4 for the EVE1 alignment, LG + F + G4 for the EVE2 alignment, and LG + F + I + G4 for the concatenated EVEs alignment according to the BIC criterion and were used to construct phylogenetic maximum likelihood (ML) trees with IQ-TREE v1.6.12 (Nguyen *et al*. 2015).

#### Flanking Region Analysis

To characterize the EVEs loci and identify potential transposable elements or other genetic features, flanking regions of the identified EVEs were extracted from the host contigs and used as BLAST queries to screen against the NCBI nucleotide (nt) and non-redundant protein (nr) databases (both accessed in October 2018).

#### Metagenomic Screening

According to the WGS screening results above, some *Flaviviridae*-related hits were detected in a shrew (*Crocidura indochinensis*) genome. However, apart from the *Crocidura indochinensis* and *Sorex araneus* complete genomes, only a limited number of shrew genomes are currently available in the NCBI WGS database. Therefore, 73 DNA experimental genomic data sets and 6 RNA-Seq transcriptome data sets (Supplementary Table S7, Supplementary Material online) from the Soricidae family were retrieved from NCBI Sequence Read Archive (SRA) database using SRA Toolkit v2.10.8 (Leinonen *et al*. 2011). Reads were mapped to the identified EVEs nucleotide references using Bowtie2 v2.3.5.1 (Langmead and Salzberg 2012). Alignment files were processed with SAMtools v1.10 (Li *et al*. 2009) and coverage was determined using bedtools v2.27.1 (Quinlan and Hall 2010) and visualized in RStudio v1.1.463.

### *In Vivo* Validation

#### Sample Collection

Based on the screening results, to further verify the presence of *Flaviviridae*-related EVEs *in vivo*, a total of 65 tissue and DNA samples from species belonging to the *Crocidura* genus and 6 other related genera of the Soricidae family, namely *Paracrocidura*, *Scutisorex*, *Suncus*, *Sylvisorex* (subfamily Crocidurinae), *Neomys,* and *Sorex* (subfamily Soricinae), were screened for the presence of the identified EVEs. These samples were previously collected in China, Vietnam, Africa, and the Eastern Mediterranean (Supplementary Table S2, Supplementary Material online) as part of other studies (Bannikova et al. 2011; Jenkins et al. 2013; Catalano et al. 2018; Van de Perre et al. 2018, 2019).

#### Target EVEs and Cytochrome b Amplification

DNA was extracted from tissue samples using the DNeasy Blood & Tissue Kit (Qiagen) following the manufacturer’s instructions.

To screen for the presence of EVEs *in vivo*, we designed 18 PCR primers (Supplementary Table S8, Supplementary Material online) spanning the 2 EVEs region and a section of the intermediate flanking region from the host genome. Amplicons were generated with DreamTaq DNA Polymerase (ThermoFisher Scientific) using the following cycling conditions: (i) 3 min of denaturation at 95°C; (ii) 35 cycles of 95°C for 30 s, 56°C for 30 s, 72°C extension for 1 min/kb; and (iii) 10 min final extension at 72°C.

To confirm the host species, and to complement available specimen information, the mitochondrial CYTB gene was amplified using general primers (Supplementary Table S8, Supplementary Material online) of the Crocidurinae subfamily. PCR reactions were conducted using the DreamTaq DNA Polymerase (ThermoFisher Scientific) with the following thermal cycling conditions: (i) 3 min of denaturation at 95°C; (ii) 35 cycles of 95°C for 30 s, 56°C for 30 s, 72°C extension for 1 min/kb; and (iii) 10 min final extension at 72°C.

All PCR products were purified using the ExoSAP-IT PCR Product Cleanup (ThermoFisher Scientific) or Zymoclean Gel DNA Recovery Kits (ZYMO Research) to remove primer dimers and unspecific products, following the manufacturer’s instructions.

#### Sanger Sequencing

The generated PCR products were sequenced by Macrogen Europe. The amplicons were mapped to the whole EVEs region (2,235 nt) from the WGS *Crocidura indochinensis* contig, and concatenated based on consensus sequence to get the complete EVEs in Geneious Prime® v2020.2.4. CYTB amplicons (∼1,140 nt) were forward and reverse sequenced and a consensus sequence was generated using Geneious Prime® v2020.2.4.

#### MinION Sequencing

For 12 samples with relatively low-quality Sanger sequencing chromatograms (additional information provided in Supplementary Table S3, Supplementary Material online), MinION sequencing was performed to obtain the complete EVEs region (∼2,235 nt) together with the CYTB gene (∼1,140 nt). The Oxford Nanopore Technologies (ONT) 1D Native barcoding genomic DNA protocol was used without the DNA fragmentation step and the barcoded amplicons were loaded onto the MinION device. We used the MinKNOW software v19.13.5 on the MinIT companion for data acquisition and basecalling. Qcat v1.1.0 (ONT, https://github.com/nanoporetech/qcat) was used to demultiplex reads under the epi2me algorithm and to trim bad quality reads and adapters with min score of 90. The EVE regions extracted from the WGS *Crocidura indochinensis* contig were used as references to map the reads with Minimap2 v.2.22 (Li 2018) using -ax map-ont parameters. Alignments were converted and indexed using SAMtools v1.10 (Li *et al*. 2009) and consensus sequences were generated using a custom Python script (Kafetzopoulou *et al*. 2019).

### Phylogenetic Analysis and Visualization

All generated EVEs sequences were translated and aligned with homologous polyproteins from available *Pestivirus* species using MAFFT v7.453 (Katoh *et al*. 2002). Sequences of dengue-2 and Zika virus (*Flavivirus* genus) were used as an outgroup. The alignment was trimmed using BMGE v1.12 (Criscuolo and Gribaldo 2010) and the filtered regions were used to construct ML phylogenetic trees using IQ-TREE v1.6.12 (Nguyen *et al*. 2015) under the best-fitting models (according to the Bayesian information criterion): LG + G4. Phylogenies were visualized and annotated using FigTree v1.4.4 (Rambaut A; http://tree.bio.ed.ac.uk/software/figtree/). Percent identity matrices were generated using Clustal Omega (Sievers *et al*. 2011) via EMBL-EBI web services (Madeira *et al*. 2019).

For species classficiation, CYTB sequences of EVEs-positive specimens (*n* = 48) were aligned using MAFFT v7.453 (Katoh *et al*. 2002) together with a data set of *n* = 393 Soricidae nucleotide sequences downloaded from NCBI. The generated alignment was trimmed in BMGE v1.12 (Criscuolo and Gribaldo 2010) and an ML phylogeny was reconstructed using IQ-TREE v1.6.12 (Nguyen *et al*. 2015) with the best-fitting model (TIM2 + F + I + G4). The phylogenies were annotated in ggtree v1.14.6 (Yu *et al*. 2017) and treeio v1.6.2 (Wang *et al*. 2020) R packages.

To investigate the co-phylogenetic relationships of our newly discovered EVEs and their hosts, we compared the topological structure between the host and EVEs phylogenies using both nuclear and mitochondrial gene information for a variable number of shrew species. For the mitochondrial gene data set, we used our generated CYTB sequences (*n* = 48), while for the nuclear gene data sets we revisited the most complete mammalian phylogeny up-to-date (Upham *et al*. 2019) and extracted genomic information for a subset of *n* = 11 shrew species on the apolipoprotein b gene (APOB) and for *n* = 10 shrew species on the breast cancer 1 gene (BRCA1). The event-based eMPRess tool (Santichaivekin *et al*. 2021) was used to assess phylogenetic congruence between the host and EVEs trees. Upon determining the most parsimonious costs for duplications, transfers and losses, we selected the representative reconciliations for the various comparisons and performed a permutation test of 100 randomizations to compute support values. Graphical representation of the congruence level between the EVEs phylogenies and the host trees was performed using the ape v5.0 (Paradis and Schliep 2019) R package.

### Characterization of Selective Pressure

We characterized the selective pressure acting on the EVE 1 and EVE 2 region. We respectively aligned the open reading frame of the complete EVE1 (318 nt) and the coding region of EVE 2 fragments (1335 nt) using MEGA v11.0.9 (Tamura *et al*. 2021). An ML tree was built based on this alignment in IQ-TREE v1.6.12 (Nguyen *et al*. 2015) under the best-fitting model HKY + F (for EVE1) and HKY + F + G4 (for EVE2). We then conducted two site-specific selection analyses to characterize the selective pressure on each site using estimates of the ratio of non-synonymous/synonymous substitution rate (*ω* = *d_N_/d_S_*): (i) FEL analyses (Kosakovsky Pond and Frost 2005) using MG94xREV model available in HyPhy software v2.5.3 (Kosakovsky Pond *et al*. 2005), and (ii) the Bayesian renaissance counting method (Lemey *et al*. 2012) implemented in BEAST v1.10.5 (Suchard *et al*. 2018). The value of *ω* quantifies the selective pressure, with *ω* > 1 suggesting positive selection, *ω* = 1 neutral evolution and *ω* < 1 negative or purifying selection.

### Divergence Time Estimation

In order to obtain an estimate of the time-scale of pestivirus evolution, we performed a molecular clock analysis of the pestivirus-EVE amino acid alignment used for phylogenetic analysis (cfr. 4.3). We used a Bayesian approach implemented in BEAST v1.10.5 (Suchard *et al*. 2018), incorporating an LG + G4 substitution model, a Bayesian skygrid coalescent prior, and an uncorrelated relaxed clock model. Following a divergence time estimate for Crocidurinae, we specify a normal prior with mean 10.8 MYA and standard deviation of 1.6 MY on the EVE most recent common ancestor. We constrained the dengue and Zika virus sequences to form an outgroup in the analyses. Markov chain Monte Carlo (MCMC) analyses were performed for 100 M generations sampling every 10,000th generation. The MCMC runs were diagnosed using Tracer ensuring that all effective sampling sizes were larger than 200.

## Supplementary Material

msac190_Supplementary_Data

## Data Availability

The data underlying this article are available in the article and in its online supplementary material. The EVE and CYTB sequences for all Crocidura species are available in the GenBank Nucleotide Database and can be accessed with the accession numbers available in Supplementary Table S3, Supplementary Material online.
