# Supplementary material for: Endogenous Viral Elements in Shrew Genomes Provide Insights into Pestivirus Ancient History
Source: Mol Biol Evol. 2022 Sep 5;39(10):msac190. doi: 10.1093/molbev/msac190 (PMC9550988; doi:10.1093/molbev/msac190)
Supplement: msac190_Supplementary_Data [file msac190_supplementary_data.zip › S_Fig1_screening_results.pdf]

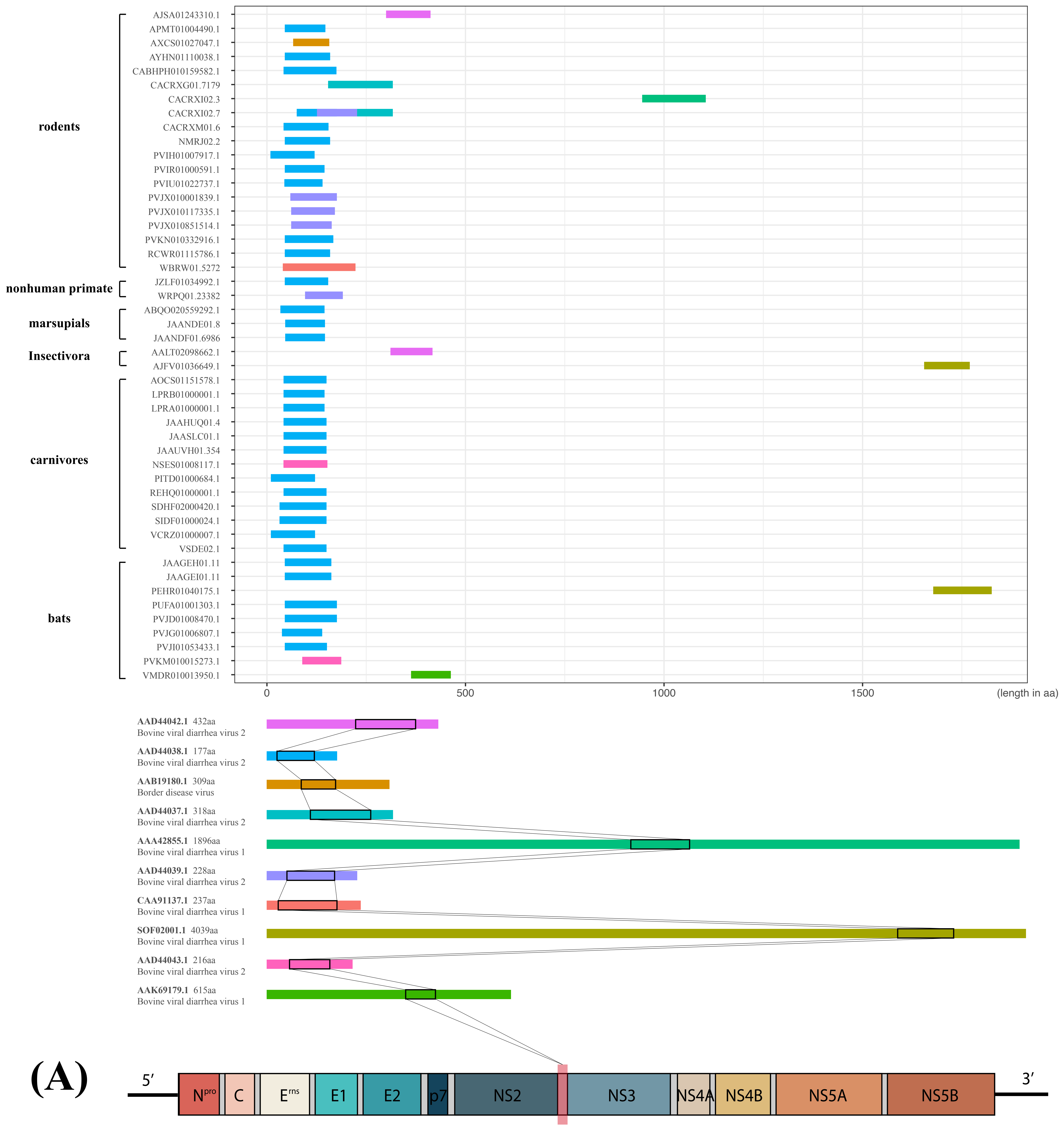

Supplementary Fig.1: (A) Positive hits result from screening against NCBI nr database and the corresponding position in specific pestiviral genome

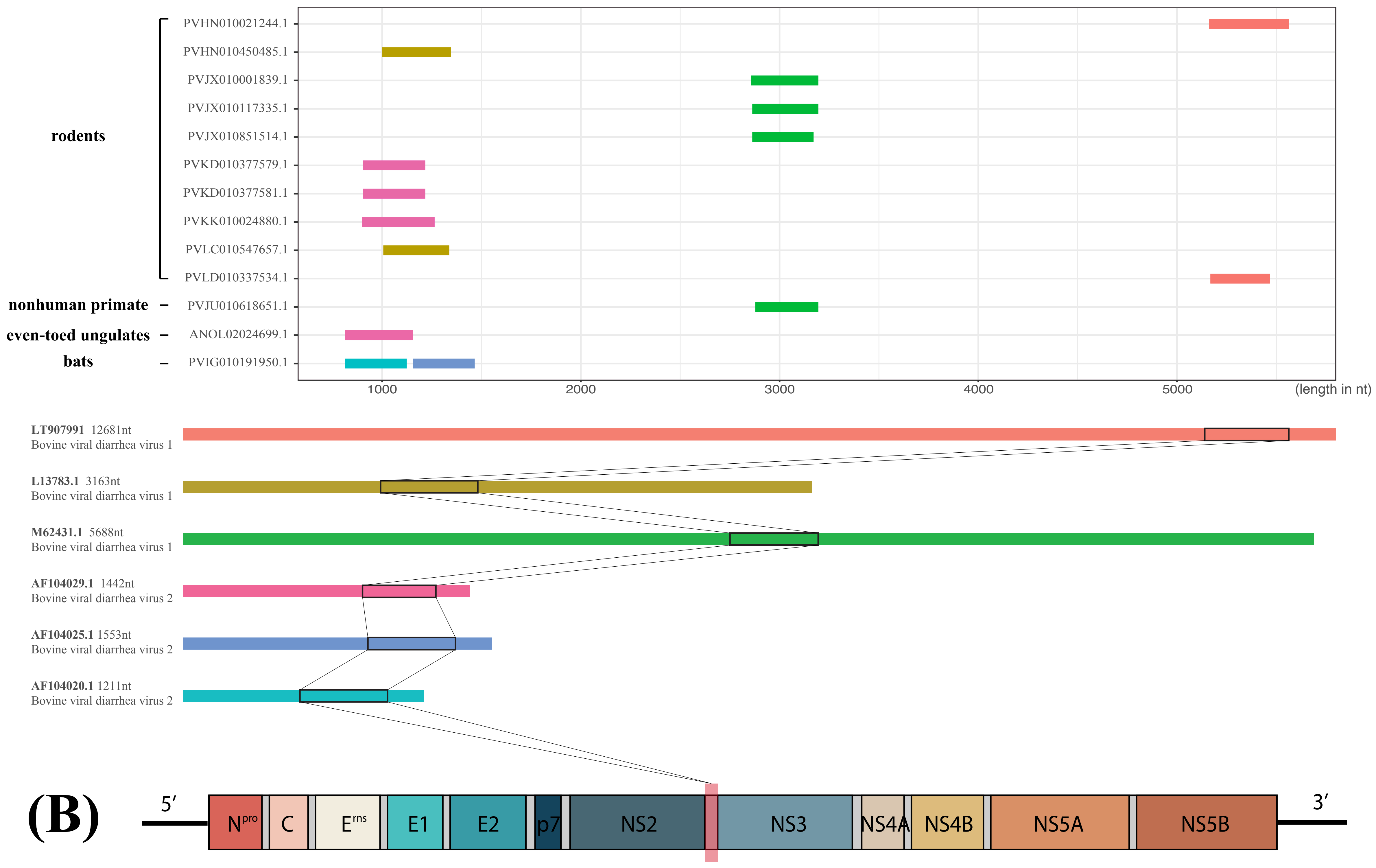

Supplementary Fig.1: (B) Positive hits result from screening against NCBI nt database and the corresponding position in specific pestiviral genome
