# Supplementary material for: Endogenous Viral Elements in Shrew Genomes Provide Insights into Pestivirus Ancient History
Source: Mol Biol Evol. 2022 Sep 5;39(10):msac190. doi: 10.1093/molbev/msac190 (PMC9550988; doi:10.1093/molbev/msac190)
Supplement: msac190_Supplementary_Data [file msac190_supplementary_data.zip › S_Fig2_C.indochinensisEVEtrees_separate.pdf]

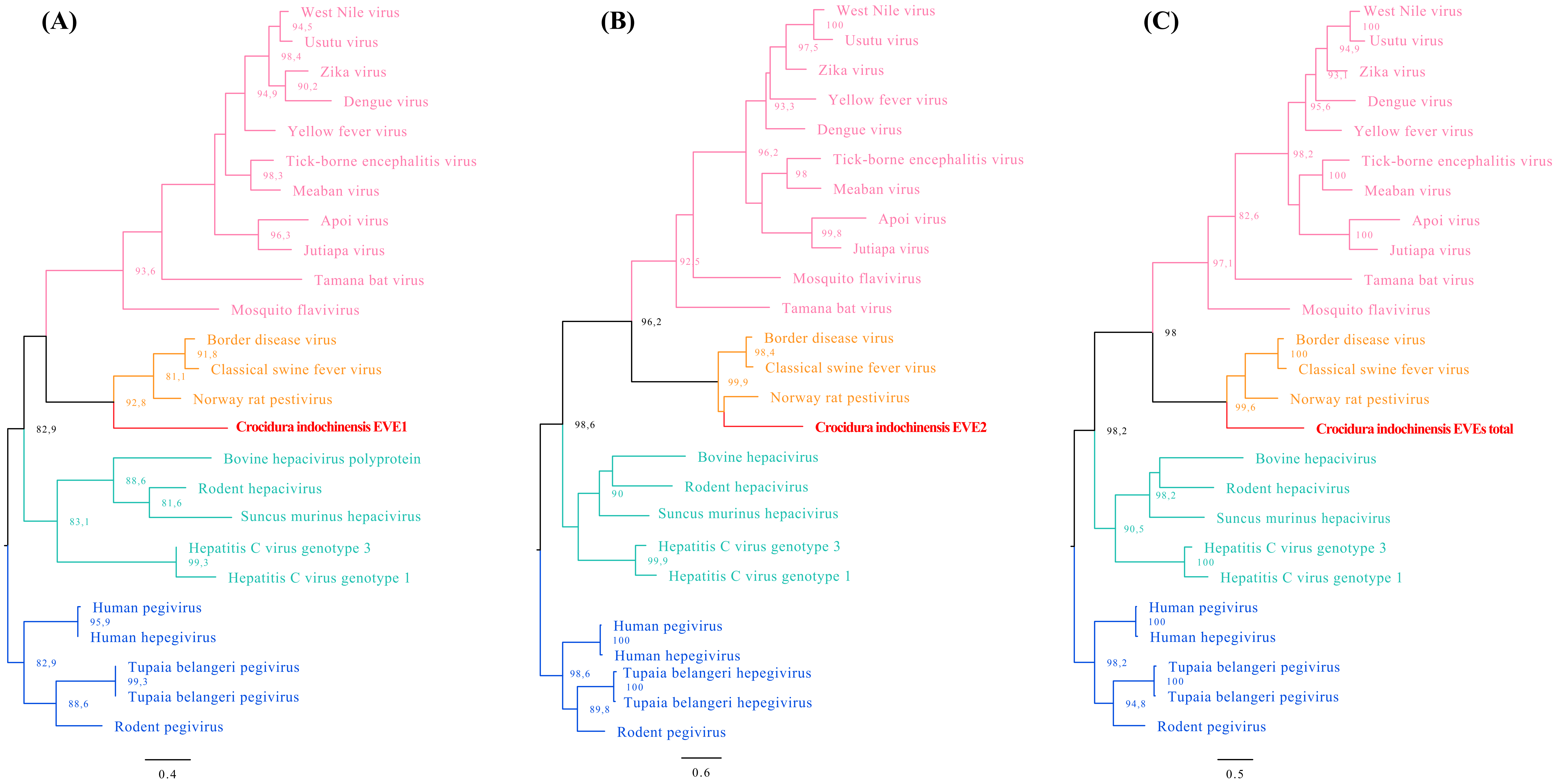

Supplementary Fig. 2: (A): Phylogenetic relationships of *Crocidura indochinensis* pesti-like EVE1 with E2 region of representative viruses from Flaviviridae family; (B): Phylogenetic relationships of EVE2 with NS2-NS3 region of representative viruses from Flaviviridae family; (C) Phylogeny based on total EVE1 and EVE2 region concatenation with E2 and NS2-3 concatenation of representative viruses from Flaviviridae family.
Clades are colored based on viral genus, pink: Flavivirus; orange: Pestivirus; green: Hepacivirus; blue: Pegivirus.
Node labels indicate Shimodaira-Hasegawa (SH)-like branch support (%, only values > 80% are shown). Scale bars indicate the number of substitutions.
