# Supplementary material for: Endogenous Viral Elements in Shrew Genomes Provide Insights into Pestivirus Ancient History
Source: Mol Biol Evol. 2022 Sep 5;39(10):msac190. doi: 10.1093/molbev/msac190 (PMC9550988; doi:10.1093/molbev/msac190)
Supplement: msac190_Supplementary_Data [file msac190_supplementary_data.zip › S_Fig3_cytb_classification.pdf]

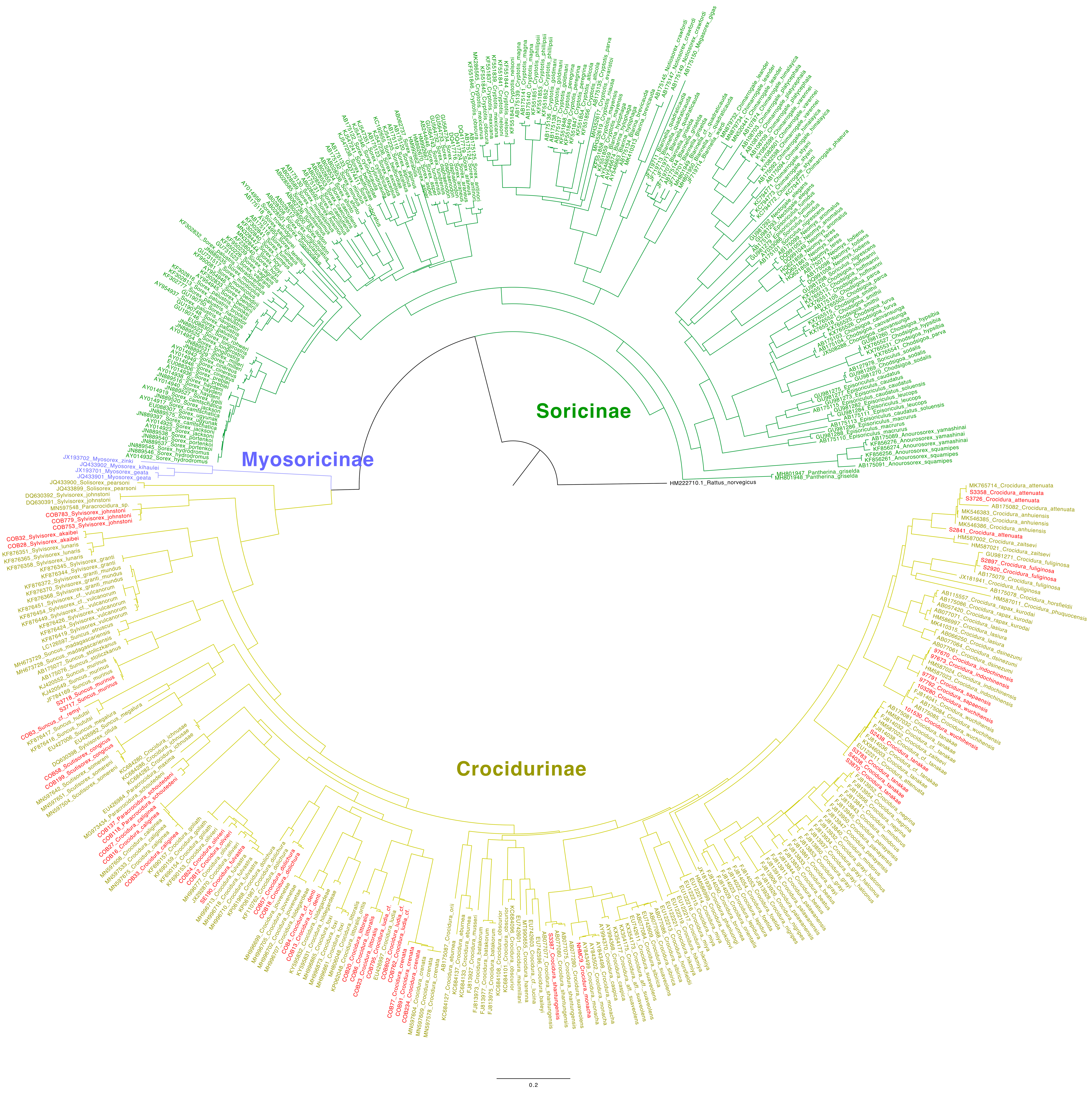

Supplementary Fig. 3: Phylogeny of current Soricidae family based on Cytb sequences. Samples detected to habour pesti-like EVEs in this study are marked in red. Scale bars indicate the number of substitutions.
