# Supplementary material for: Endogenous Viral Elements in Shrew Genomes Provide Insights into Pestivirus Ancient History
Source: Mol Biol Evol. 2022 Sep 5;39(10):msac190. doi: 10.1093/molbev/msac190 (PMC9550988; doi:10.1093/molbev/msac190)
Supplement: msac190_Supplementary_Data [file msac190_supplementary_data.zip › S_Fig4_EVE1&2_pestivirus.pdf]

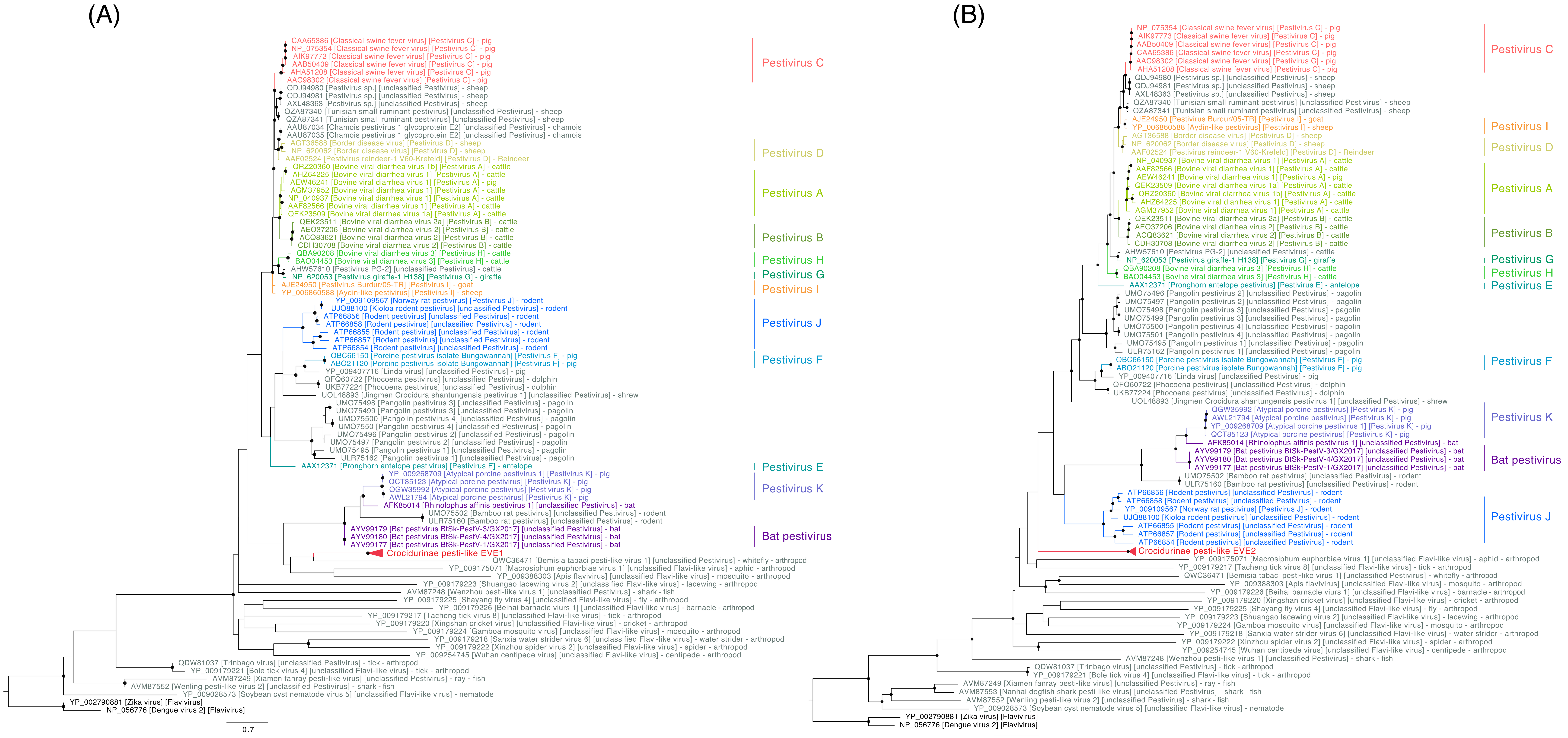

Supplementary Fig. 4: (A) Phylogenetic relationships of pesti-like EVE1 region with E2 region of representative Pestiviruses (B) Phylogenetic relationships of pesti-like EVE2 region with NS2-NS3 region of representative Pestiviruses. Dengue and Zika virus (Flavivirus) were used as outgroup. Clades are colored based on viral species. Nodes labled in black circles indicate Shimodaira-Hasegawa (SH)-like branch support (%, only values > 80% are shown). Scale bars indicate the number of substitutions.
