# Supplementary material for: Endogenous Viral Elements in Shrew Genomes Provide Insights into Pestivirus Ancient History
Source: Mol Biol Evol. 2022 Sep 5;39(10):msac190. doi: 10.1093/molbev/msac190 (PMC9550988; doi:10.1093/molbev/msac190)
Supplement: msac190_Supplementary_Data [file msac190_supplementary_data.zip › S_Fig5_tanglegrams.pdf]

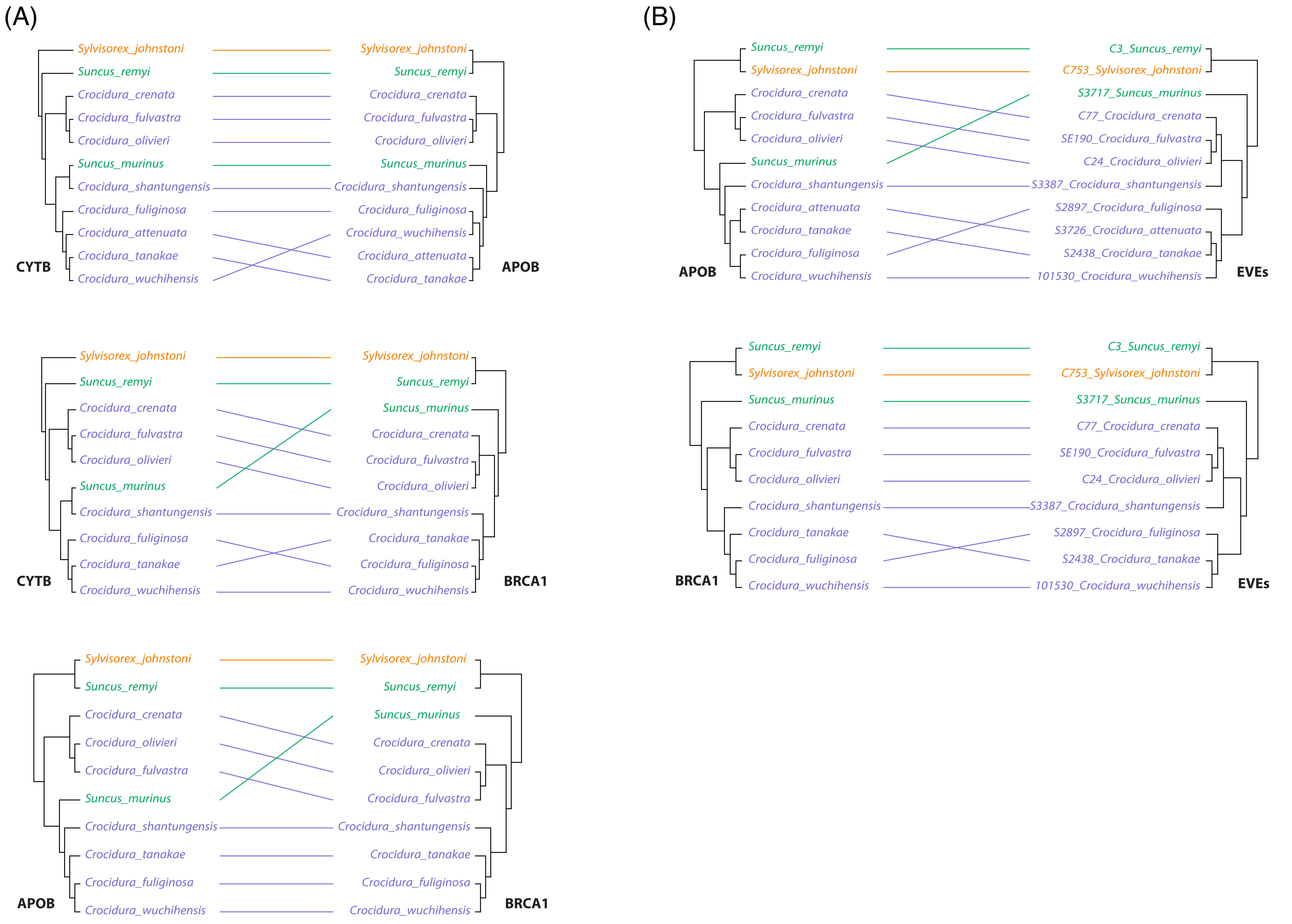

Supplementary Fig. 5:
(A) Tanglegrams of two nuclear genes (APOB, BRCA1) and one mitochondrial gene (CYTB) phylogenies for the available shrew species;
(B) Tanglegrams of two nuclear genes (APOB, BRCA1) with EVEs phylogenies for the available shrew species.
The clades were colored by shrew genus, orange: *Sylvisorex*; green: *Suncus*; purple: *Crocidura*.
